# Exploration of family caregivers’ experiences on coping in dementia care in Ghana: a phenomenological study

**DOI:** 10.1186/s40359-024-01862-y

**Published:** 2024-06-20

**Authors:** Precious Adade Duodu, Joshua Okyere, Bibha Simkhada, Ransford Akrong, Caroline Barker, Warren Gillibrand, Padam Simkhada

**Affiliations:** 1 Department of Nursing, School of Human and Health Sciences, University of Huddersfield, Queensgate, Huddersfield, West Yorkshire, England, UK; 2 Department of Population and Health, University of Cape Coast, Cape Coast, Ghana; 3 School of Nursing and Midwifery, College of Health Sciences, Kwame Nkrumah University of Science and Technology, Kumasi, Ghana; 4 Educational Assessment and Research Center, Osu, Accra Ghana; 5 School of Human and Health Sciences, University of Huddersfield, Queensgate, Huddersfield, West Yorkshire, England, UK

**Keywords:** Alzheimer’s, Family caregiver, Dementia, Coping, Phenomenology, Qualitative research

## Abstract

**Background:**

Dementia is an important public health and geriatric concern for sub-Saharan African countries, including Ghana. Evidence shows that persons living with dementia are often supported and cared for by family caregivers in the community. In the execution of these services to the persons living with dementia, family caregivers are overwhelmed and experience heightened stress that results in serious repercussions. Therefore, the aim of this study was to explore family caregivers’ experiences on coping in dementia care in Ghana.

**Methods:**

Adopting a descriptive phenomenological design, individual face-to-face interviews were conducted among thirty unpaid family caregivers of persons living with dementia in Ghana. Semi-structured interview guides were used. The data analysis process followed Clarke and Braun’s framework analysis.

**Results:**

Six themes were generated from the textual data. These themes were captioned as: (1) empathy and perspective-taking; (2) family support and cohesion; (3) coaxing and pampering of persons living with dementia; (4) humour and positive communication; (5) spiritual support; and (6) ethical/moral consideration in dementia caregiving.

**Conclusion:**

We conclude that unpaid family caregivers of persons living with dementia in Ghana adopt varied strategies to cope with the strains of caregiving. Healthcare facilities that provide services to persons living with dementia could incorporate caregiver preparatory training or education for family caregivers. This training should focus on briefing family caregivers about the potential strains that they are likely to encounter. Also, the training could focus on equipping family caregivers with the knowledge and skills to effectively communicate and care for the persons living with dementia using person-centered approaches. Key stakeholders such as the Ghana Health Service and Alzheimer’s Ghana must raise awareness about the dangers of caregivers’ violation of the autonomy and freedom of persons living with dementia as they navigate through the challenges of caregiving. Lastly, faith-based institutions need to be considered as key stakeholders in dementia interventions since they could play a critical role.

**Supplementary Information:**

The online version contains supplementary material available at 10.1186/s40359-024-01862-y.

## Background

Dementia, an umbrella term for several neurocognitive diseases such as Alzheimer’s disease, is considered an important public health concern, particularly of older adults due to the strong association between age and dementia [1]. Global evidence suggests that dementia is increasing among the older populations [2]. In the past, many believed that dementia was a problem for the high-income or developed countries; however, recent report indicates that developing countries such as countries in sub-Saharan Africa (SSA) face a high burden of dementia [3]. Evidence indicates that each year, there are over 367,000 new cases of dementia reported in SSA; it is estimated that the prevalence of dementia in the region would increase to 3.48 million in 2030 and surpass 7.62 million cases by 2050 [3].

Dementia is characterized by significant impairment in cognitive functioning [4]. These impairments often manifest in the form of “prolonged deterioration, during which persons with dementia lose their mental, cognitive, and motor abilities” [5]. In the advanced stages of the disease, the person living with dementia is unable to speak or move about freely [6, 7]; thus, limiting their movements and independence to perform tasks. Consequently, many persons living with dementia depend on caregivers to go about their activities of daily living.

Literature shows that persons living with dementia are often supported and cared for by family caregivers in the community [8]. For instance, reports in the United States of America indicate that unpaid caregivers offer approximately 17.0-18.6 billion hours of care to persons living with dementia at an estimated economic value of $232–244 billion [9, 10]. As indicated, persons living with dementia suffer cognitive dysfunction that affects their independence to complete daily tasks [6, 7]. Hence, family caregivers play an important role in managing the activities of daily living such as assisting persons living with dementia to attend healthcare appointments, providing the financial resources needed for their upkeep, running errands, bathing, and managing their diets, among others [11–13]. In the execution of these services to persons living with dementia, family caregivers are overwhelmed and experience heightened stress that results in serious repercussions.

A study conducted in Ghana has shown that family caregivers often experience stigma from the public [14]. Likewise, Nguyen et al. [15] and Flemons et al. [16] have revealed that family caregivers of persons living with dementia experience multiple strains including competing time demands, loss of income, poor sleep quality, poor eating practices, social isolation, and psychological distress (i.e., anxiety, frustration, depression, etc.). These strains that characterize the caregiving of persons living with dementia negatively impact the quality of life and wellbeing of family caregivers. Hence, there is a need to understand how family caregivers navigate through or cope with these challenges.

Coping, in this context, refers to “constantly changing cognitive and behavioural efforts to manage specific external and/or internal demands that are appraised as taxing or exceeding the resources of the person” [17]. Evidence from other jurisdictions such as the Germany [18] and Israel [19] indicate that family caregivers adopt a plethora of coping mechanisms that can be broadly categorized as either problem-focused, emotion-focused, or dysfunctional coping strategies. According to Roache et al. [18], the problem-focused coping strategies aim at directly reducing the stressor, utilizing approaches like active coping, planning, and seeking instrumental support. Conversely, emotion-focused coping involves altering the relational meaning of the stressor rather than directly changing it, incorporating methods such as acceptance, seeking emotional support, humour, positive reframing, and turning to religious coping whereas the dysfunctional coping strategy is often characterized by behaviours such as disengagement, denial, distraction, self-blame, substance use, and venting, which may not effectively address the underlying stressor and could exacerbate the situation [18]. However, in the context of Ghana, it is unclear how family caregivers cope with the stress and burden of caring for persons living dementia.

The Ghanaian kinship and family is such that it is difficult for family members to reject caregiving roles [20]. Thus, familial care is ascribed to individuals as responsibility to members of the family who are with a health condition [21]. Despite the cultural reinforcement of caregiving as a responsibility that must be honoured by healthy family members to the sick, it fails to adequately prepare individuals to assume caregiving roles and also does not provide any sort of mechanisms to cushion caregivers when they begin to face challenges in the performance of their duty. Family caregivers’ adoption of healthy coping strategies is critical to their quality of life [22]. Yet, to the best of our knowledge, there is currently no published research that has explored the coping strategies of family caregivers of persons living with dementia in Ghana. This knowledge gap makes it difficult to develop policies and programmes to support family caregivers of persons living with dementia. Against this background, the present study aimed to explore unpaid family caregivers’ experiences on coping in dementia care for persons living with dementia in Ghana.

## Methods

### Study design

To align with the study’s objective, we adopted a qualitative research methodology, specifically opting for a descriptive phenomenological approach. This research design emphasizes the exploration and representation of the lived experiences of individuals, rather than solely relying on subjective interpretations of a particular phenomenon [23]. Descriptive phenomenology was considered the most suitable research design for our study because it aimed to uncover the first-hand experiences of unpaid family caregivers in relation to the strategies that they adopt to cope with the strains of caring for persons living with dementia.

### Study settings

This study recruited participants from eight health facilities, namely Ejisu Government Hospital; Juaben Government Hospital; University Hospital, Kwame Nkrumah University of Science and Technology (KNUST); Kumasi South Hospital; Manhyia District Hospital; Onwe Government Hospital; Kumasi Cheshire Home; and Tafo Government Hospital.

### Participant selection and sampling

We employed a purposive sampling technique to select and enlist unpaid family caregivers as participants. Family caregivers accompanying their patients clinically diagnosed with dementia to seek care at the hospitals were recruited for the study. Given the intricate nature of caregiving and our specific interest in investigating the coping strategies adopted from the standpoint of unpaid family caregivers, purposive sampling enabled us to focus on a deliberate selection process. This method guaranteed that participants had direct engagement and profound understanding of the caregiving experience, thereby enhancing the richness and genuineness of the collected data [24, 25].

To be eligible for participation in the study, participants had to meet the following inclusion criteria: (a) be recognized as an unpaid family caregiver of a person living with dementia (i.e., a family member, such as a spouse, adult child, or other close relative, who takes on the role of coordinating and managing the daily care needs of the PwD on a consistent and ongoing basis); (b) engage in face-to-face interactions with a person living with dementia at least once a week over the last six (6) months; (c) the individual must be providing emotional care, instrumental care or both to the person living with dementia; and (d) be an adult aged 18 years or older. Any individual who did not meet all these criteria was excluded from the study.

### Data collection procedures

We utilized a semi-structured interview guide as our primary data collection tool. This guide was developed based on existing literature related to caregiving for persons living with dementia, shaping the formulation of both key questions and probing inquiries [14, 16, 18]. The interview guide covered questions such as: (a) Could you please describe your experience with dementia? (b) What strategies do you adopt to cope with the challenges of caring for your relative who lives with dementia? (c) Which of the coping strategies have worked well or not worked well in managing the challenges of caring for your relative with dementia?

All individual interviews were conducted face-to-face at the various healthcare facilities. Initially, we recruited research assistants who were qualified nurses with bachelor’s degrees and provided them with comprehensive training over two weeks. These nurses were all indigenes of the Ashanti Region and were proficient in speaking, writing, and understanding Twi (the dominant local language of the Ashanti Region of Ghana). The two-week training covered topics such as the study’s purpose and objectives, the data collection tool, ethical considerations, and the screening and selection of unpaid family caregivers as participants. Subsequently, we assigned these research assistants to their respective healthcare facilities. The data collection period was 17th April to 31st May 2023.

During the first week of data collection, the research assistants identified and screened potential participants. Unpaid family caregivers identified during this initial week were provided with detailed information about the study, its procedures, benefits, and their rights and responsibilities if they chose to participate. In the following week, the research assistants followed up with these unpaid family caregivers to obtain their responses. All thirty unpaid family caregivers that were screened agreed to participate in the study. The research assistants then coordinated interview times with the caregivers. All participants agreed to conduct the interviews at the healthcare facility where they were recruited. The interviews were conducted in both English and Twi, based on the preference of the participants. Specifically, twenty-one of the interviews were conducted in English while the remaining nine were in Twi. Importantly, none of the participants withdrew from the study, and the interviews were audio-recorded, typically lasting an average of forty-five minutes each.

### Data management and analysis

The data analysis process followed Clarke and Braun’s thematic analysis framework [26]. Our decision to go by framework analysis rather than grounded theory was premised on the point that we were not interested in developing or generating theories from the data. Rather, we sought to explore and understand the lived experiences of caregivers. This method allows for systematic categorization and interpretation of data according to key themes relevant to the caregivers’ coping strategies.

Initially, the audio data were transcribed verbatim. The interviews, originally in Twi, were translated by the data collectors. To ensure translation accuracy, a professional translator proficient in Ghanaian languages reviewed the transcripts against the audio recordings. The completed translations received validation from PAD, JO, and RA. Subsequently, JO commenced the formal analysis, with assistance from PAD and RA. The transcripts were read multiple times to become familiar with the data. Subsequently, the transcripts were imported into QSR NVivo-12 for effective data management and coding. Within the transcripts, key phrases, statements, and expressions relevant to the caregiving experience were identified, and initial codes were assigned [26]. These initial codes were then used to create condensed meaning units, capturing the core essence of participants’ descriptions and emotions. The assigned codes were further reviewed and organized into themes that reflected common aspects of the caregiving experience. Following this, the authors engaged in discussions to elaborate and refine the themes, aiming to create clear and comprehensive descriptions that effectively conveyed the depth and breadth of participants’ experiences. This validation process continued until a consensus was reached regarding the final themes. The research findings were presented by including selected excerpts from the transcripts to illustrate and support the identified themes.

### Rigour and trustworthiness

To establish credibility, we engaged in repeated readings and immersed ourselves deeply in the narratives provided by the participants [27]. Also, using bilingual researchers helped to confirm the quality of the translation. Furthermore, we exclusively utilized verbatim quotes to illustrate the viewpoints of the participants. We ensured transferability by furnishing a comprehensive description of our research methodology and the study’s geographical context [28]. As a result, researchers exploring similar settings can draw upon our methodology for guidance and replication. Throughout the data collection process, we conducted debriefing sessions with our research assistants. This proactive step aided in clarifying any potential inconsistencies and served to prevent any introduction of bias by the interviewers, thereby contributing to the achievement of confirmability, transferability, authenticity, and dependability. Additionally, we meticulously maintained records of the audio data and transcripts to bolster the confirmability of our research.

### Ethical considerations

Ethical approval from the Ghana Health Service Ethics Review Committee (GHS-ERC) [ID Number: GHS-ERC: 005/02/23] including approvals from the respective health directorates of the sampled healthcare facilities and permission from all the healthcare facilities were obtained to recruit participants for this study. An approval was also obtained from the School Research Ethics and Integrity Committee (SREIC), University of Huddersfield, United Kingdom (SREIC Reference: SREIC_ExtApp_2023_001). Additionally, oral and written informed consent was obtained from each participant. For those who had no formal education, their rights and responsibilities were read to them and interpreted in the language preferred by each participant (either Twi or English) to gain their informed consent. Participants were assured of confidentiality, data protection, anonymity, and the right to withdraw from the study without any consequence, loss of benefit, care or treatment to them or their relatives with dementia in the facilities where they were recruited. The study was performed in accordance with the ethical standards as laid down in the 1964 Declaration of Helsinki and its later amendments or comparable ethical standards.

## Results

### Socio-demographic characteristics

There were thirty (30) unpaid family caregivers in this study, comprising of 8 (26.7%) males and 22 (73.3%) females. The age categories were 25–34 years (30%), 35–44 years (20%), and 45 years or older (50%). Regarding their highest educational level, there were none (6.7%), Junior High School (20.0%), Senior High School (20.0%), and tertiary (53.3%) qualifications. For marital status, there were single (40.0%), married (16.7%), and divorced (43.3%) participants. Daughters (53.3%) and sons (23.3%) of persons living with dementia were the dominant family caregivers (see supplementary file for details).

### Unpaid family caregivers’ experiences on coping in dementia care

**Fig. 1:**
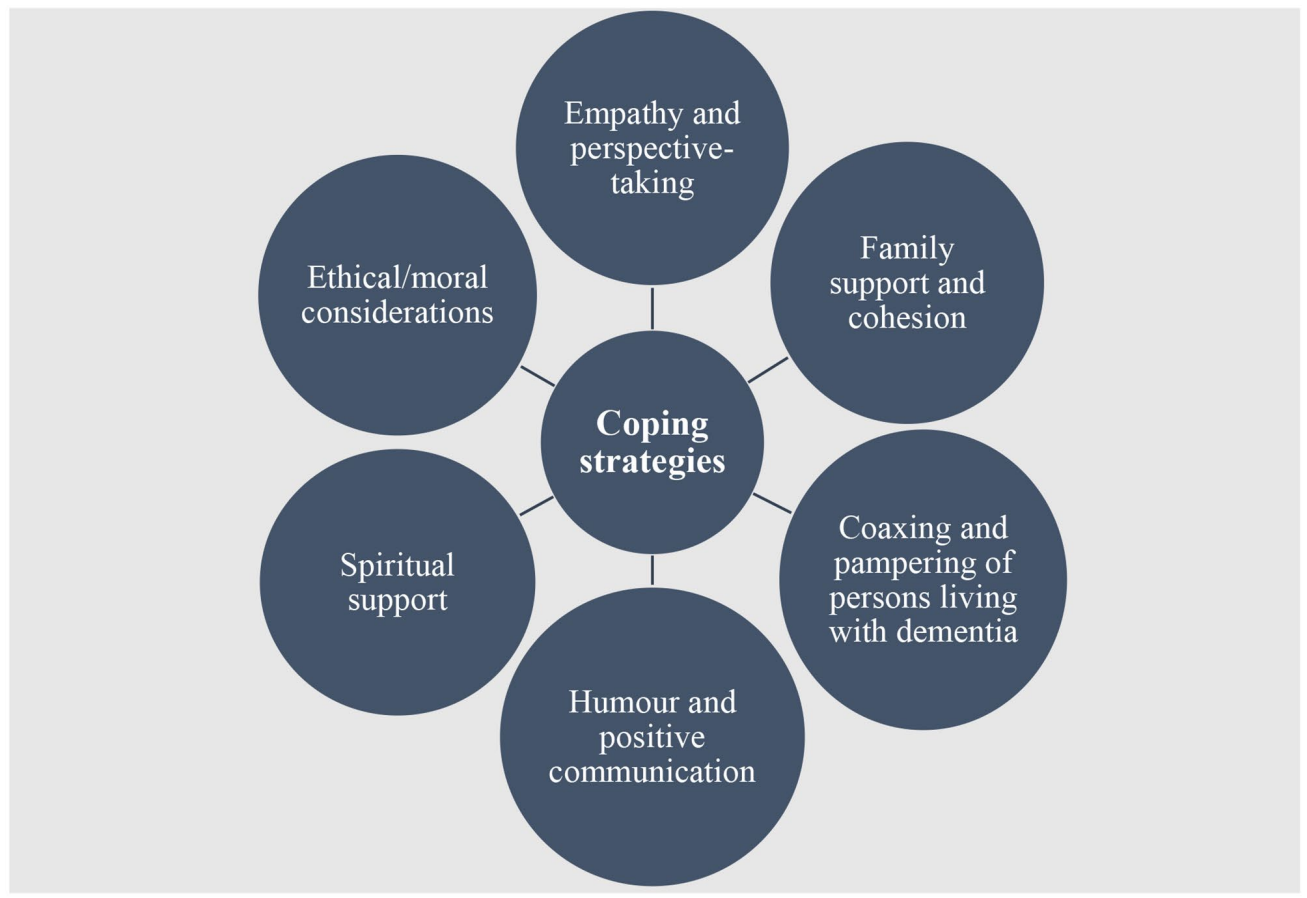
Emerging themes

Six themes were generated from the textual data. These themes were captioned as: (1) empathy and perspective-taking; (2) family support and cohesion; (3) coaxing and pampering of persons living with dementia; (4) humour and positive communication; (5) spiritual support; and (6) ethical/moral consideration in dementia caregiving (see Fig. 1).

### Empathy and perspective-taking

The analysis revealed that empathy and perspective-taking was a common strategy adopted by unpaid family caregivers to cope with the strains of caregiving. Particularly, family caregivers asserted that the persons living with dementia often behave in ways that deviate from normal. This tends to result in agitation, anger, and frustration on the part of the unpaid family caregivers. However, in such instances, they empathize with the persons living with dementia and try to rationalize why that individual is behaving in a deviant way. The family caregivers ascribed to the perspective that dementia is part of the ageing process, therefore, they can only be patient and empathize with the persons living with dementia. This is what some unpaid family caregivers had to say:

*“I always tell her that even we who are young fall ill sometimes and it resolves with time. At your age, all your blood vessels are obstructed; they are not like ours. The way blood circulates freely in me is not the same way in her body so she should pray. She attends CAC [a Christian denomination] so she should continue to pray to God that ‘if it’s time for you to call me [to eternity], please do but if it’s not, give me the strength to continue’. So, when I come to her, all I do is to encourage her.”* (50-year-old son of person living with dementia attending Onwe Government Hospital).

*“Let me say that if I am in a situation of anger, normally I use to ask myself why this guy is doing that, but after a few minutes I will say to myself that it’s because of his situation that is why he is doing that. So, I take it like that.”* (31-year-old son of person living with dementia attending Manhyia Government Hospital).

Another family caregiver who perceived that her father’s condition would have been difficult but for her positive perspective and approach to his care. She narrated that:

*“There is this kind of thing he used to do outside the house; urinating or putting up a lot of, let me say, unhuman behaviours. With our situation, maybe in a different house they would have thrown him somewhere or maybe they won’t even look after him again but because of the kind of patience I have, the situation has improved.”* (28-year-old daughter of person living with dementia attending Tafo Government Hospital).

### Family support and cohesion

Unpaid family caregivers of persons living with dementia identified the support of their family members as a resource that helped them to cope with the strains and challenges of caregiving. According to the narrative from the participants, there has been a strong bond between members of the family. This ensures that every family member contributes to the caregiving process. Hence, if the primary family caregiver is absent, another family member is able to step in and assume the role as a caregiver. A family caregiver shared this experience:

*“He is our father so when his relapse starts, any of us comes to help him. Even if we are asleep and another is awake, he that is awake calls for us that Baba’s condition has started. We all come together and help him. When I am away, my siblings are the ones who take care of him. No one speaks to him impolitely or harshly because we know it is a sickness and also that was not how he was at first.”* (42-year-old daughter of person living with dementia attending Ejisu Government Hospital).

Another family caregiver narrated:

*“The family tries to support; for those that are not here would call and ask of her. She could mention her children’s names and we would call them for her to say good morning. She is able to respond to greetings and salutations. Her grandchildren sometimes come around to talk to her even though she would not understand. Her loved ones also pass by sometimes.”* (60-year-old daughter of person living with dementia attending Kumasi South Government Hospital).

It was evident from the analysis that the financial burden that often confronts unpaid family caregivers was ameliorated because of the family cohesion and support. The family pooled financial resources to support the person living with dementia, thereby reducing the burden on the primary family caregiver. This is reflected in the quote below:

*“I do not have any financial challenges when it comes to her because my brothers send money every week. Those in Accra [capital city of Ghana] send theirs every month, so when it comes to struggling financially, that is not the case.”* (40-year-old daughter of person living with dementia attending Kumasi South Government Hospital).

A few family caregivers experienced abandonment. Some recounted moments where all other family members had neglected caregiving responsibilities, leaving them to bear the burden alone. This lack of support did not only add to their emotional and physical strain but also hindered their ability to provide optimal care for their loved ones. Despite these challenges, the caregivers who had a strong support system were able to navigate through the difficulties more effectively and provide better care for their family members in need.

“*People used to ask me where her children are, since I had gone for her to take care of her as an in-law. I did not like the way her children treated her, especially how they spoke to her so I went for her*.” (41-year-old daughter-in-law of person living with dementia attending Onwe Government Hospital).

### Coaxing and pampering of persons living with dementia

The study revealed that family caregivers struggled with ensuring that their family members who were living with dementia adhered to treatment or medications. To deal with this challenge, family caregivers adopted the strategy of coaxing and pampering of persons living with dementia to get them to adhere to the medications. This finding is exemplified by the following narrations:

*“Sometimes, she would say she does not like medications made in Ghana so I would have to connive with someone that the medications are imported and say flattery words to her such as the medications will make her look young and pretty.”* (46-year-old son of person living with dementia attending Kumasi South Government Hospital).

*“We take it easy with her. I make sure to be patient whenever she relapses or refuses to take her medications. I have also encouraged her to take the medications for she will be cured when she consistently takes them.”* (45-year-old daughter of person living with dementia attending Tafo Government hospital).

Unpaid family caregivers asserted that the strategy of coaxing and pampering was effective, especially in situations where the persons living with dementia refuses to comply with whatever suggestions and instructions given to them. In such cases, getting angry as a caregiver does not yield the desired outcome. Rather, being patient and pampering resulted in the desired outcome.

*“Oh! We are all humans so you need patience and pampering, if you do not pamper or flatter her, you will not succeed with her. You should not get angry when dealing with her. I sometimes wrap my hands around her and pamper her”* (60-year-old daughter of person living with dementia attending Kumasi South Government Hospital).

### Humour and positive communication

Unpaid family caregivers described the use of positive comments and encouragement as effective strategies for addressing moments of sadness or distress in caring for persons living with dementia. Additionally, family caregivers mentioned the use of a good sense of humour as a way to alleviate distress and improve the mood of persons living with dementia. By introducing lighthearted or amusing elements into their interactions, caregivers could redirect the focus of persons living with dementia from distressing thoughts to more positive and engaging experiences. One participant shared this:

*“Sometimes, I pass funny comments when I see her sad. Whenever I see her sad, I could see that she is thinking a lot and would be making some tragic story about her situation, but I would always encourage her with some positive words that all will be fine, and highlight that some other people’s situations are even worse than hers.”* (37-year-old daughter of person living with dementia attending Juaben Government Hospital).

Other family caregivers expressed that:

*“I exhibit a positive attitude towards him when I realize that he is desperate. I treat him the normal way either in the presence or absence of family members… The communication is the best strategy that has worked so far; from my observation, whenever he is lonely and you approach him with a conversation, he feels relieved. Paying attention and showing care are also some of the strategies that works.”* (34-year-old daughter-in-law of person living with dementia attending Ejisu Government Hospital).

*“One of my sisters calls and speaks with her, and it calms her when she is being aggressive…. I call my brothers in Accra and they speak with her, it calms her down; they also make promises to move her to Accra. One of my sisters promises to visit her and then she calms down”.* (40-year-old daughter of person living with dementia attending Kumasi South Government Hospital)

### Spiritual support

Unpaid family caregivers of persons living with dementia relied on faith as a coping strategy emphasized the concept of divine timing. They understood that healing, whether physical or spiritual, unfolds according to God’s plan. This perspective provided them with patience and resilience in the face of the uncertainties associated with caring for persons living with dementia. This acceptance brought a sense of peace and resignation, allowing primary caregivers and family members to cope with the emotional challenges of caring for persons living with dementia with a sense of surrender to a higher authority.

*“Nobody is more powerful than God, and He has told us to ask Him for everything, so I think it is just a matter of time; God Himself will heal her in His own time. When the time comes for her to be healed, she will be healed. If she dies, we know it is also the doing of the Lord. So, I don’t get worked up over the problems I experience and the financial difficulties. I know that in time, everything will work out for my good.”* (42-years-old daughter of person living with dementia attending Ejisu Government Hospital).

Another family caregiver shared similar perspective:

*“I used to worry a lot about what might happen to my mother. Her condition was deteriorating. I worried so much that I even became sick. Can you imagine? Me, who is supposed to care for her – I ended up being sick. But I came to the realization that it is all the doing of the Lord. God is the one who allows things to happen, for His own reasons. So, these days, I no longer get worried. I am challenged to do my best for her. God will take care of the rest.”* (48-year-old son of person living with dementia attending Ejisu Government hospital).

A few family caregivers further took action to seek help from divine healers by taking the persons living with dementia to prophets, pastors, and other divine healers. For some, the objective was to be certain about whether or not the disease was caused by some spiritual forces or not. They believed that seeking spiritual intervention could provide answers and potentially offer a solution to the challenges they were facing. This act of seeking divine help also provided them with a sense of hope and comfort, knowing that they were doing everything they could to alleviate the suffering of their loved ones.

“*Everyone has their spiritualist and we contacted a few about what was happening to her. Some of my neighbours who she physically abused even took her to a pastor to pray for her. A few pastors even came around to pray for her.” (*46-year-old son of person living with dementia attending Kumasi South Government Hospital).

“*At first, we could take her to my prophet for him to give us herbs for us to use to bath her. When you do that, it disappears from her and she becomes normal.”* (53-year-old daughter of person living with dementia attending Manhyia Government Hospital).

### Ethical/moral considerations in coping with dementia care

This theme delves into the intricate ethical dilemmas faced by family caregivers as they navigate the realm of dementia care. It sheds light on the daily challenges caregivers encounter, as they grapple with maintaining the wellbeing of their loved ones while honouring the ethical imperative to preserve dignity and autonomy. Caregivers candidly shared their experiences, unveiling the nuanced complexities inherent in their roles. They recounted instances where difficult decisions had to be made, such as the necessity of restricting the movements of their family members with dementia to prevent wandering. The caregivers recounted the moral tensions surrounding the act of locking doors to confine individuals with dementia indoors. While undertaken with the intention of ensuring safety, such measures prompt reflections on the delicate balance between safeguarding and respecting autonomy. This is what one participant had to say:

*“I used to lock her indoors when she was with me so that I could take the children to school to prevent her from going outside to get lost. So, I would come back to find her banging the door and I think it is because she felt oppressed.”* (49-year-old son of person living with dementia attending Kumasi South Government Hospital).

Similarly, another caregiver recounted the ethical quandary of administering medication to a resistant family member, grappling with the tension between ensuring medical adherence and respecting individual preferences.

*“She did not like taking the tablets so I would have to grind them and mix with water and force it down her throat … I had to force her to give her the medications.”* (41-year-old daughter-in-law of person living with dementia attending Onwe Government Hospital).

## Discussion

Previous studies exploring the experiences of executing caregiving roles to persons living with dementia [14, 16, 18] have shown that family caregivers face innumerable challenges ranging from financial strains, psycho-emotional distress, stigma, stress, and burnout. Therefore, understanding how family caregivers of persons living with dementia cope with these challenges would provide valuable insights to inform the development and implementation of holistic programmes for family caregivers. Hence, we explored unpaid family caregivers’ experiences on coping in dementia care for persons living with dementia in Ghana. Overall, our findings resonate with previous studies [18, 19] that have argued that family caregivers adopt either problem-focused (in this case: coaxing and pampering of persons living with dementia), emotion-focused (empathy and perspective-taking; spiritual support; humour and positive communication) or dysfunctional coping strategies (i.e., the ethical violation of the autonomy and movement of persons living with dementia).

This study revealed that unpaid family caregivers adopted different strategies of empathizing and taking a positive perspective. We argue that empathy and perspective-taking have a positive impact on the caregiver-patient relationship. When family caregivers approach their role with empathy, patience and understanding, it fosters a sense of trust and emotional connection. This, in turn, creates an enabling environment for improved communication and more harmonious caregiving dynamics. Our findings align with Lloyd et al. [29] whose study revealed that self-compassion acts like empathy significantly reduce the caregiver burden among dementia caregivers. Additionally, we found that some family caregivers further used avoidant coping mechanisms to demonstrate their compassion for their persons living with dementia. Even though family caregivers employ this strategy to aid their patients, research has shown that avoidant coping mechanisms, such as denial or withdrawal, may temporarily alleviate caregiver burden but can lead to long-term negative consequences [30, 31]. It is crucial for healthcare professionals to provide support and education to caregivers on more adaptive coping strategies that promote their own wellbeing while providing compassionate care for their loved ones with dementia.

Our findings suggest that family support and cohesion play an important role in helping family caregivers of persons living with dementia to cope with the strains of caregiving. Similar findings have been reported in Brazil [32] and Japan [33]. Family caregivers often bear the primary responsibility for the care of persons living with dementia. However, in situations where there is a strong bond and support system within the family, caregivers are better equipped to handle the emotional, physical, and financial strains associated with caregiving. The emotional support from family members contributes to caregiver resilience while the financial support alleviates the financial burden often placed on the primary family caregiver [34].

Extant literature shows that non-adherence to medications among persons living with dementia is a common phenomenon [35, 36]. However, participants of the present study overcame this challenge by gently persuading persons living with dementia to take their medications. Caregivers adapted their communication styles to the unique needs and preferences of the persons living with dementia. Furthermore, the strategy of coaxing and pampering aligns with the principles of patient-centered care, where the preferences and emotional wellbeing of the persons living with dementia are central to the caregiving approach [37].

We found that unpaid family caregivers of persons living with dementia adopted humour and positive communication as a coping strategy, which is consistent with previous literature [38, 39]. A plausible explanation for this emotion-focused coping strategy is that it provides a means for family caregivers to regulate their own emotions and maintain a positive attitude in the face of challenging situations [40]. The adoption of this coping strategy is rooted in the Ghanaian culture where the people are conditioned to remain happy amidst challenges. For instance, the Ghanaian adage that says that ‘even when we cry, we stop to sneeze’ and ‘a person in debt must still eat’. These proverbial sayings within the Ghanaian culture emphasize the need to adopt positivity in the face of any challenge, including in situations of strains from caregiving.

The study also revealed that family caregivers relied on religious or spiritual reinforcements or support as a coping strategy. This is consistent with a plethora of studies [41–43] that have found that religious reinforcements serve as a major coping mechanism against the numerous strains of caregivers. Caregivers who sensed a strong spiritual connection within themselves and in their relationship with the Divine conveyed an enhanced ability to care for both themselves and their loved ones with dementia. The study revealed that this coping strategy enabled caregivers to exercise patience as they navigated through the stress and strains of caregiving. Similar to McGee et al.’s study [43], our study demonstrates that caregivers used spirituality as a resource to confirm the etiology of their family member’s condition. Essentially, looking up to spiritual support or divine interventions offers solace and a sense of purpose, thereby helping caregivers to navigate the emotional challenges associated with dementia care.

This study also revealed situations where family caregivers adopted dysfunctional coping strategies. Specifically, we found that some caregivers applied force and adopted maladaptive strategies such as keeping persons living with dementia behind locked doors. The findings concur with Spittel, Maier and Kraus’s study [44] that found that caregivers in Ghana tend to put persons with mental disorders and dementia behind locked doors. It is imperative to acknowledge that placing individuals with dementia in such restrictive environments raises serious human rights concerns, as it infringes upon their freedom and dignity. This practice, although observed in a subset of caregivers in our study, is ethically and socially problematic. Hence, there is a need for targeted interventions aimed at reshaping caregiver approaches and establishing ethical guidelines.

### Implications for policy and practice

Our findings underscore a need to prioritize the implementation of comprehensive education and awareness initiatives tailored to equip families with the necessary skills and knowledge to effectively navigate the complexities of dementia caregiving. These initiatives should encompass various aspects, including understanding the progression of dementia, managing behavioural and psychological symptoms, ensuring safety in the home environment, and facilitating effective communication with individuals living with dementia. Additionally, these educational interventions must go beyond imparting practical caregiving skills to equip caregivers with strategies to manage challenging behaviours without resorting to restrictive measures, fostering a supportive and empowering environment for both caregivers and those receiving care. Also, psychosocial support services must be readily accessible, offering counselling, support groups, and respite care to alleviate caregiver stress and prevent burnout, thereby enhancing the quality of care provided while safeguarding caregivers’ mental health. This study also highlights the significance of integrating spiritual care into dementia caregiving practices. Caregiver training programmes in Ghana must also focus on enhancing caregivers’ spiritual coping mechanisms. These initiatives should be designed to cater for caregivers of diverse cultural and religious backgrounds, respecting and integrating various spiritual traditions and beliefs. Such trainings must emphasize activities such as meditation, prayer sessions, and discussions on faith-related topics, aimed at helping caregivers deepen their spiritual connections and resilience.

### Strengths and limitations

Utilising a descriptive phenomenological design proved advantageous for our research, enabling us to comprehensively capture the real-life experiences of unpaid family caregivers of persons living with dementia. However, it is crucial to acknowledge certain limitations. Firstly, our study exclusively focused on unpaid family caregivers of persons living with dementia who sought care at the specific healthcare facilities where our study was conducted. Consequently, the insights gained may not fully represent caregivers whose care recipients did not engage with any of the healthcare facilities included in our study. Additionally, there exists the potential for self-reported bias, given that participants were recounting their personal lived experiences. The study did not differentiate the type of dementia that family caregivers were rendering services to. As such, we are unable to tell whether coping strategies identified would be ubiquitous across all types of dementia.

## Conclusion

We conclude that unpaid family caregivers of persons living with dementia in Ghana adopt varied strategies to cope with the strains of caregiving. Healthcare facilities that provide services to persons living with dementia could incorporate caregiver preparatory training or education for family caregivers. This training should focus on briefing family caregivers about the potential strains that they are likely to encounter. Also, the training could focus on equipping family caregivers with the knowledge and skills to effectively communicate and care for the persons living with dementia using person-centered approaches. Key stakeholders such as the Ghana Health Service and Alzheimer’s Ghana must raise awareness about the dangers of caregivers’ violation of the autonomy and freedom of persons living with dementia as they navigate through the challenges of caregiving. Finally, while enacting and implementing effective national policies to improve the care of persons living with dementia and their families, faith-based institutions need to be considered as key stakeholders in dementia interventions since they could play a critical role.

### Electronic supplementary material

Below is the link to the electronic supplementary material.

Supplementary Material 1

## Data Availability

All relevant data are within the paper. Any other data or material associated with this manuscript are available on request through the corresponding author (PAD).

## Abbreviations

GHS-ERC: Ghana Health Service Ethics Review Committee
KNUST: Kwame Nkrumah University of Science and Technology
SREIC: School Research Ethics and Integrity Committee SSA: Sub-Saharan Africa
