# Supplementary material for: Exploration of family caregivers’ experiences on coping in dementia care in Ghana: a phenomenological study
Source: BMC Psychol. 2024 Jun 20;12:361. doi: 10.1186/s40359-024-01862-y (PMC11191216; doi:10.1186/s40359-024-01862-y)
Supplement: Supplementary file 1 — Supplementary Material 1 [file 40359_2024_1862_MOESM1_ESM.docx]

**Supplementary file: Sociodemographic characteristics of study participants**

| **Characteristics** | **Sample size (N = 30)** | **Percentages** |
| --- | --- | --- |
| ***Sex***  Male  female | 8  22 | 26.7%  73.3% |
| ***Age (in years)***  *2*5-34  35-44  ≥45 | 9  6  15 | 30%  20%  50% |
| ***Highest educational level***  None  Junior High school  Senior High School  Tertiary | 2  6  6  16 | 6.7%  20%  20%  53.3% |
| ***Marital status***  Single  Married  Divorced | 12  5  13 | 40%  16.7%  43.3% |
| ***Relationship with PwD***  Daughter  Son  Daughter-in-law  Niece  Wife  Sister  House help | 16  7  2  1  2  1  1 | 53.3%  23.3%   6.7%  3.3%  6.7%  3.3%  3.3% |
